# Ability of Laying Hens to Distinguish Between Companions According to Their Success in Gaining Access to Food

**DOI:** 10.3389/fvets.2018.00234

**Published:** 2018-10-01

**Authors:** Anette Wichman

**Affiliations:** Department of Animal Environment and Health, Swedish University of Agricultural Sciences, Uppsala, Sweden

**Keywords:** laying hens, social cognition, learning, foraging behavior, informed decision

## Abstract

Laying hens (*Gallus gallus*) are social birds with cognitive abilities related to having a functional interaction with their peers. Gaining knowledge about for example new food sources from other individuals can be a valuable complement to individual learning and probably even more so if one copies the behavior of successful individuals. In this study the aim was to investigate if a bird would identify another bird as being successful at gaining access to food. A social cognition feeding test was developed where birds could move freely together between several scattered food sources. Two different methods were used for training. In method 1, the observer hens were exposed to a skilled demonstrator hen that gained access to the food sources and an unskilled demonstrator hen (that gained no access to food) at the same time when trained together in a trio. In method 2, the observer was trained in two different pair constellations, with a skilled and unskilled demonstrator, respectively. In the test situation for both methods birds were paired, one observer was tested once with the skilled demonstrator and once with the unskilled demonstrator. Observations of how much the observer birds followed the two different demonstrators to the food sources, although no food was available during testing, were carried out. Observers trained in trios (method 1) did not show any difference in following behavior between the skilled and unskilled demonstrator, but observers that had been trained in pairs (method 2) showed more following behavior toward the skilled demonstrators than the unskilled demonstrator (*P* = 0.005). Thus the results indicate that laying hens are able to use another bird as a cue of whether they will get access to food.

## Introduction

Living together with conspecifics can provide many benefits of which one could be the increased possibility to exchange information about important resources between individuals (1). It has been suggested that individuals should be selective with whom they copy [i.e., do something in the same way as someone else) and when depending on the circumstances (2). One social strategy could be to copy successful individuals (3) and thus it should be advantageous for individuals to be able to acquire knowledge about skills or experiences that specific individuals possess. The aim of this study was to investigate if laying hens distinguish between different individuals depending on these hens' previous ability to lead them to food. The observer birds' behavior would thus not be based on these birds' ability to get access to food at the time, but on their previous experience of these birds' ability to do so and provide indications of whether they use the other hens as social cues and know whom it should be most beneficial to follow.

Hens have evolved as a species living in small groups of individuals (4). These groups are often organized in dominance hierarchies which are based on individual recognition of the other members of the group (5). There is some evidence that hens are able to make indirect inferences about dominance relationships by observing interactions between other hens (6), which implies that hens have fairly developed social-cognitive skills. Both theoretical models and empirical findings suggest several different strategies exist for when social learning occurs depending on e.g., the species, situation, and environment (7). For example size could be an indicator of previous success and nine-spined sticklebacks (*Pungitius pungitius*) have been found to rather copy the patch choice of large demonstrators compared to small demonstrators (8). Similar to this reasoning, it could be assumed that more dominant individuals are generally successful in life and thus a strategy to copy more dominant individuals could be in line with a strategy to copy more knowledgeable individuals (9, 10). There is some evidence that dominance seems to influence social learning in chickens (10). However, when (11) compared the effect of social learning of laying hens depending on if the demonstrators had shown prior foraging success or not, no difference was found in the behavior of the observers. The prior success was induced by letting the demonstrator feed in a separate pen while being observed by the other hens and then the influence of social learning was tested in a separate key-pecking task. In comparison, Quelea birds (*Quelea quelea*) which were allowed to interact together have been suggested to find the location of resources they are in need of by the presence of knowledgeable flock companions (12). This indicates that the ability to interact with the demonstrator could have affected the learning process. These Quelea birds use of the other birds as demonstrators seemed to be based only partly on that they followed these birds to the food source since it happened that naïve observer birds went first to the resource. Thus, their behavior could be based on reading cues from the other birds or some other mechanism and not on previous knowledge that the demonstrators were apt at leading them to food. Also guppies have been found to follow other guppies more that had been trained to move toward a previously known food source (13) and there are indications that bats can gain access to food by following other bats (14). Giraldeau and Lefebvre (15) investigated how a flock of pigeons learned a food opening technique and found that a few pigeons were responsible for solving the task and discovered the food (producers) whereas the other pigeons took advantage of this (scroungers). They observed the distance between the birds and found that during a session the scroungers tended to be found more frequently in the area nearby the producer than in areas with only other scroungers. Whether this was because the scroungers had identified the individual producer from previous knowledge of this individual's behavior or because of how the producer behaved during that session is unclear.

Held et al. (16) observed interactions between two pigs searching for food in an arena. One was an informed pig which had knowledge about where the food was hidden and one was an uninformed pig which did not have this knowledge but was larger than the other pig. They found that the larger uninformed pigs that could displace the other smaller pigs were able to use the knowledge of another pig to get access to a food source. Held et al. (16) called this the informed forager paradigm that one individual with a greater competitive ability can benefit by other individuals having knowledge about where to find a resource. Since the procedure was repeated several times the dominant pigs were able to change tactics from searching for food themselves at random and instead follow a subordinate pig which was informed about where the food was located. Similarly as for the Quelea birds (12) there was no specific investigation in this study of whether the pigs knew beforehand that another individual was an “informed forager.” The focus was more on the connection between following or exploitation of the other pig's knowledge and based on that individual's actions at the time. However, in a follow up study where subdominant pigs were revisiting food sources on repeated occasions either alone, with a scrounging dominant pig or together with a non-scrounging dominant pig their behavior was adjusted depending on the situation. Right from the start of the test the pigs behaved more similar when alone and together with the non-scrounging companion compared to when they were together with the scrounging individual. This suggested that the pigs had learnt that these two pigs to which it was familiar behaved differently and therefore adjusted its tactics accordingly (17).

The theory that it should be advantageous for animals to some extent base their decision on whom to copy based on whether this individual is successful (18) thus has support based on cues such as behavior, size, and dominance both at the time and at least for some species like pigs on previous knowledge of specific individuals.

Natural feeding behavior in feral fowl consists of birds in a flock walking around searching for feed like seeds, fruit, insects etc. (19). They often synchronize their behavior and stay within sight or hearing distance of each other and if one animal discovers an attractive source of food the others quickly try to join in McBride et al. (4). The availability of different food sources will vary over time and season (19) and thus it is possible that some hens have more experience of different food sources and where to search. The hypothesis in this study is that hens are able to learn that some individuals are more reliable food finders compared to other birds and themselves, and thus follow and approach these birds more in a situation where they can search for food compared to birds that have been unskilled in finding food. This knowledge should be based on the demonstrator birds‘ previous success of finding food and not their success in the test situation. A test was developed that allowed hens to interact together and the observer bird was able to eat together with the skilled food finding bird during training and thus get the direct benefit from following its companion to a food source.

## Materials and methods

### Ethical note

The study was approved by the Swedish animal research ethics committee C250/10.

The study contained only behavior observations and tests and no invasive procedures were performed. The animals' health was checked at least once per day. Birds from batch 1 moved to a private person after their participation in the study and batch 2 were culled. Culling method used was stunning with a blow to the head, quickly followed by dislocation of the neck.

### Animals and housing

Two batches of commercial laying hens were used in the study in two separate time periods. The first batch consisted of 60 Lohmann Selected Leghorn (LSL) hens and the second batch of 64 Bovans Robust. Both batches were purchased from breeders and had been reared in floor systems and arrived at the experimental facilities (Lövsta research station, Uppsala) at 16 weeks of age. They were housed in eight pens with initially seven or eight birds per pen. The birds were individually marked with leg rings in different colors. A few hens were culled before the experiment started in the first batch. One hen had a prolapse, three were subjected to cannibalism and one was a suspected cannibal. In the second batch, one hen was culled due to prolapse during the experiment. The pens were 1.15 × 1.50 m and contained wood shavings as litter, nest boxes and a perch (1.15 m long and 52 cm high). Water and conventional feed was provided ad lib. Lights were on from 7 to 19 and temperature was kept around 20 degrees.

The number of agonistic interactions performed between individuals in each group was observed when birds were 18–26 weeks of age for batch 1 and from 20 to 25 weeks of age for batch 2. Both aggressions performed and received were observed on an individual level for 250 min in batch 1 (10 min of observations/group on 25 different occasions in the home pen) and for 240 min for batch 2 (20 min of observations per group on 6 occasions in home pen and 6 occasions in an external arena). Birds were then ranked according to the ratio between aggressions performed and received and the birds selected to participate in the test were the individuals that were neither ranked as the most dominant nor subdominant individuals in each group. No detailed evaluation of the rank order between the participating individuals was possible due to too few scored interactions between birds.

### Social learning study

#### Arena design

**Figure 1:**
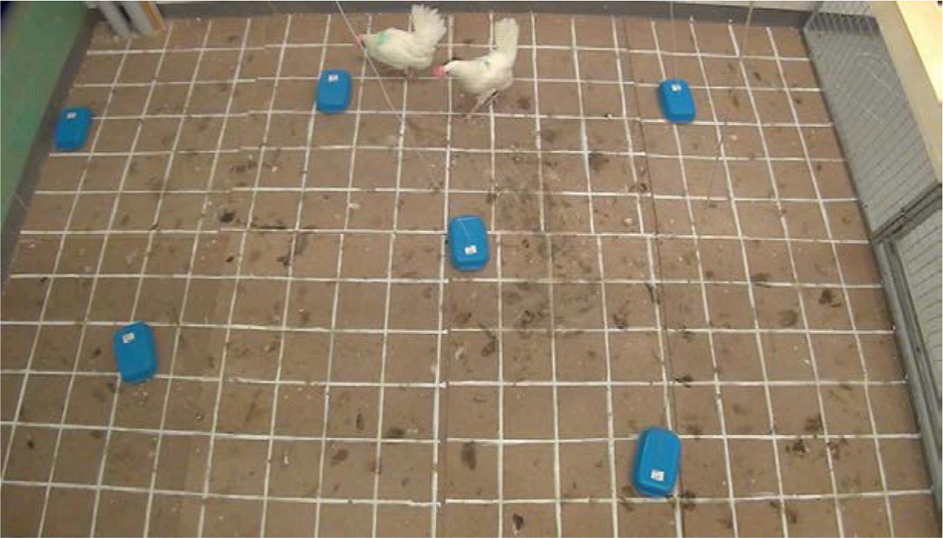
The arena where the social cognition feeding test was carried out. Six bowls with lids that could be opened from the outside were placed on the floor.

The aim of the social cognition feeding test was that it would resemble a natural situation where birds could move around and search for food where one of the birds could be selected to be a skilled demonstrator that finds food that the observer can share. The arena where the training and testing was carried out was 3.6 × 2.4 m and placed in an adjacent room to where the birds were housed. Two of the sides of the arena were made up by the walls of the room and the other two sides were made of net (2.0 m high). The floor was covered with wooden boards with a painted grid pattern (20 × 20 cm). Six bowls with lids were spread out on the floor in the arena (Figure 1). Each bowl contained corn (approx a spoonful) and the lid could be opened by the human experimenter from outside the arena by a rope attached to the lid. This person sat on a chair outside the arena in full view of the hens and opened the lid of a food bowl when the selected skilled hens approached or pecked a bowl.

Both the training and testing were filmed and the hens were marked with paint on their back in blue, black, green, or red to provide for individual identification. There were no indications that the paint had any adverse effect on the birds. The birds that were to be trained or tested were carried to the test arena together in a large plastic container right before being released in the arena.

#### Training

The training method was modified between the two batches. Birds in the first batch were trained in trios, whereas birds from the second batch were trained in pairs where each bird participated in two different pair constellations.

For batch 1, training started when the birds were 37 weeks of age and three birds from each of the eight pens took part in the experiment (*N* = 24). One of the birds was a skilled demonstrator and the other two birds doubled as each one of them were both an unskilled demonstrator and an observer.

The training of the birds in batch 2 started when they were 36 weeks of age. Four birds per pen were used in the test (*N* = 28). Two of the birds were demonstrators and two were observers. One bird became ill near the end of the training period and therefore only birds from seven of the eight pens were tested.

##### Demonstrator training

In batch 1, one of the birds in each trio was selected to be the skilled demonstrator bird. The other two birds in the trio functioned as unskilled demonstrators and as observers for each other (Table 1). The unskilled demonstrator was used as a control to compare if the observer could differentiate between this bird and the skilled demonstrator that got access to the feed. Initially, the selected skilled demonstrator bird was trained separately to feed from bowls containing corn in the arena on one occasion. Subsequently, on two consecutive days the skilled demonstrator bird was trained with another bird (which did not participate in the test later on) to feed in the arena.

**Table 1:** Overview of the roles of the individuals and how they were combined in the training and testing sessions in batch 1 and 2.

**Individuals**	**Ability to find food**	**Training**	**Testing**	
**Batch 1**				
D = Demonstrator	Skilled	In trio 7 times	D+ O1	Food pair
O1 = Observer /Demonstrator	Unskilled	D + O1 +O2	O1 + O2	Non-food pair
O2 = Observer /Demonstrator	Unskilled		D + O2	Food pair
**Batch 2**		In pairs, 7 times/pair	
D1 = Demonstrator	Skilled	D1 + O1	D1 + O1	Food pair
	Unskilled	D1 + O2	D1 + O2	Non-food pair
D2 = Demonstrator	Skilled	D2 + O2	D2 + O2	Food pair
	Unskilled	D2 + O1	D2 + O1	Non-food pair
O1 = Observer				
O2 = Observer				

In batch 2 the two designated demonstrators from one pen were habituated together to eat corn from the bowls in the arena. In general most birds were motivated to feed off the corn but some individuals were probably inhibited by being in the test arena. In batch 1 three demonstrators were exchanged with new ones from the same group in the initial stages of training because they did not feed reliably from the food bowls and in batch 2 a few birds were exchanged already during habituation.

##### Observation sessions

Each trio in batch 1 was habituated together in the test arena on 2 consecutive days when no food was provided. The actual training was conducted once per day on 7 consecutive days, where the food bowls were opened when the skilled demonstrator bird approached them and made the food accessible for all the birds. During each training session the trio remained in the arena for a maximum of 10 min or were removed earlier if the skilled demonstrator bird had approached all bowls and “opened them.”

In batch 2 out of the four birds from one pen, two were designated as demonstrator birds and two as observers. The demonstrator birds were paired with each one of the two observers and acted as a skilled demonstrator with one observer (food pair), and as an unskilled demonstrator with the other observer (non-food pair, Table 1). Thus, as in the situation with trios in batch one, an observer bird could observe one skilled and one unskilled demonstrator, but in this case they observed them at different times rather than together since birds were tested in pairs and not in trios.

**Figure 2:**
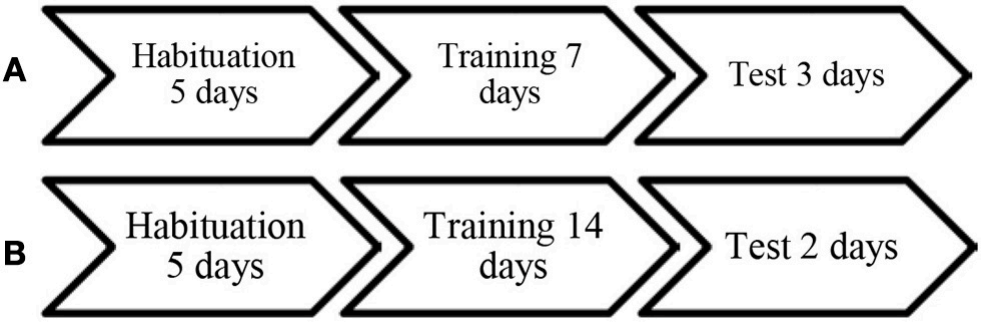
Timeline of training and testing of the two batches, **(A)** batch 1 and **(B)** batch 2.

All birds in batch 2 were trained once per day, seven times in each pair constellation, thus each bird was trained 14 times in total (Figure 2). During training in the food pair all the bowls contained corn and during training in the non-food pairs the bowls were empty and the lids remained closed. In the first and second day of training, all birds were trained in their food pair (demonstrator is skilled at getting access to food) but after that the training of food and non-food pairs was varied between days. During one training session pairs remained in the arena for a maximum of 7 min. A food pair was removed earlier if they had already finished all corn. The maximum time was reduced from 10 min that was used for the first batch. The reason for this was that most of the activity, both the opening of the food bowls by skilled demonstrators, and unsuccessful attempts to find food by unskilled demonstrators had finished by this time. Thus if they remained for a longer time in the arena the demonstrators were no longer demonstrating their ability to find food. The number of training sessions was set to be equal for all birds in the same batch. In the final training sessions in batch 1 all trios opened all bowls. In batch 2, in the final training sessions 10 pairs had opened and finished all food bowls, 2 pairs opened 5 bowls, and the remaining 2 pairs opened 4 and 3 bowls respectively.

#### Testing

Testing began the day after training finished for both batches. During testing, the food bowls were empty and remained unopened for all pairs during all testing. The pairs were in the arena for 3min. For batch 1 the birds were tested in pairs and each bird was tested twice, once with each one of the other birds from the same trio.

The birds in batch 2 were tested in the same way where each observer bird was tested once in its food pair and once in its non-food pair. Order of testing was varied between pens (half the pens were in their food pairs the first test day and in their non-food pairs the second test day and vice versa for the other pens).

#### Behavior observations during testing

Continuous observations of the number of times each individual bird performed a behavior directed toward the food bowls, or toward the other bird such as following it or had an aggressive interaction with it (Table 2) were carried out. The same behaviors were observed for all the birds irrespective of whether they were demonstrators or observers.

**Table 2:** Behavior observations carried out during testing of the birds in the social feeding test.

**Referred to in results as**	**Behaviors**	**Description**
Peck and approach bowl (combined approach and peck bowl)	Approach bowl	Walk up toward bowl (stopping) with head over or just next to bowl, only scored for the bird that approaches a bowl first.
	Peck bowl	Peck at bowl or rope, counted as one peck per visit to bowl (not the amount of pecks to the bowl at the same visit).
Follow bird (combined follow and approach bird)	Follow bird	Walk clearly in the direction of the other bird when this individual is moving. Walks at least five consecutive steps and get one score for each new five steps.
	Approach bird	Get one score if it walks five steps or more toward the other bird when this one is standing still so that it comes within one bird length distance to the other bird.
Aggressive	Aggressive	Attack or peck at the head of the other bird.
Avoid	Avoid	Crouching behavior, move away from aggressive attack or clearly avoid getting close to other bird.

### Statistical analyses

All analyses were carried out in IBM SPSS statistics 22. Data was not normally distributed; therefore, the difference between the birds' behaviors toward the two different individuals it was paired with was analyzed using a Wilcoxon Signed Rank test in which each bird was its own control. For comparisons between categories of birds (such as skilled vs. unskilled) the Mann–Whitney *U*-test was used.

Since the skilled demonstrator in batch 1 had been changed in three of the trios during training, all analyses were carried out where all birds from the eight trios were included and with separate analyses where only the birds from the five trios where the participating birds had remained unchanged from the start were included. Only for the parameter “following behavior by demonstrators” did this affect whether or not there was a significant difference between the compared groups. Therefore, only the more conservative results including the five non-manipulated trios are presented here, with the exception of the results for this specific parameter where both analyses are reported.

## Results

### Pecking and approach behavior toward bowls

**Figure 3:**
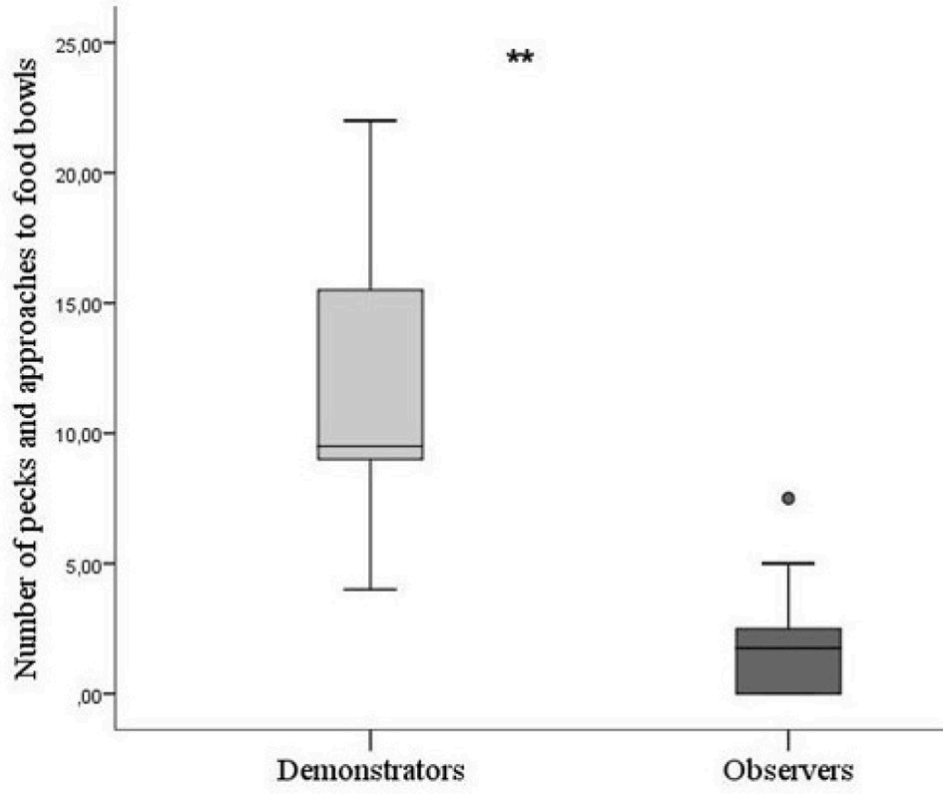
Number of pecks toward food bowls by birds in batch 1. Based on the mean of the two times one bird was tested in each of its pairs. Thick horizontal lines indicates median nr of pecks, boxes span from the first to the third quartiles and the whiskers represent 95% confidence interval; outliers are denoted by dots. Demonstrators (*N* = 5) are the light gray bar and observers (*N* = 10) dark gray bar (five groups included). The asterisks indicate a significant difference between treatments.

There was a significant difference between skilled demonstrators and observers in batch 1 during testing in how often they pecked at and approached the unopened bowls. Skilled demonstrators pecked and approached significantly more than observers (Mann–Whitney *U-*test; *P* = 0.003, *N* = 15, Figure 3).

**Figure 4:**
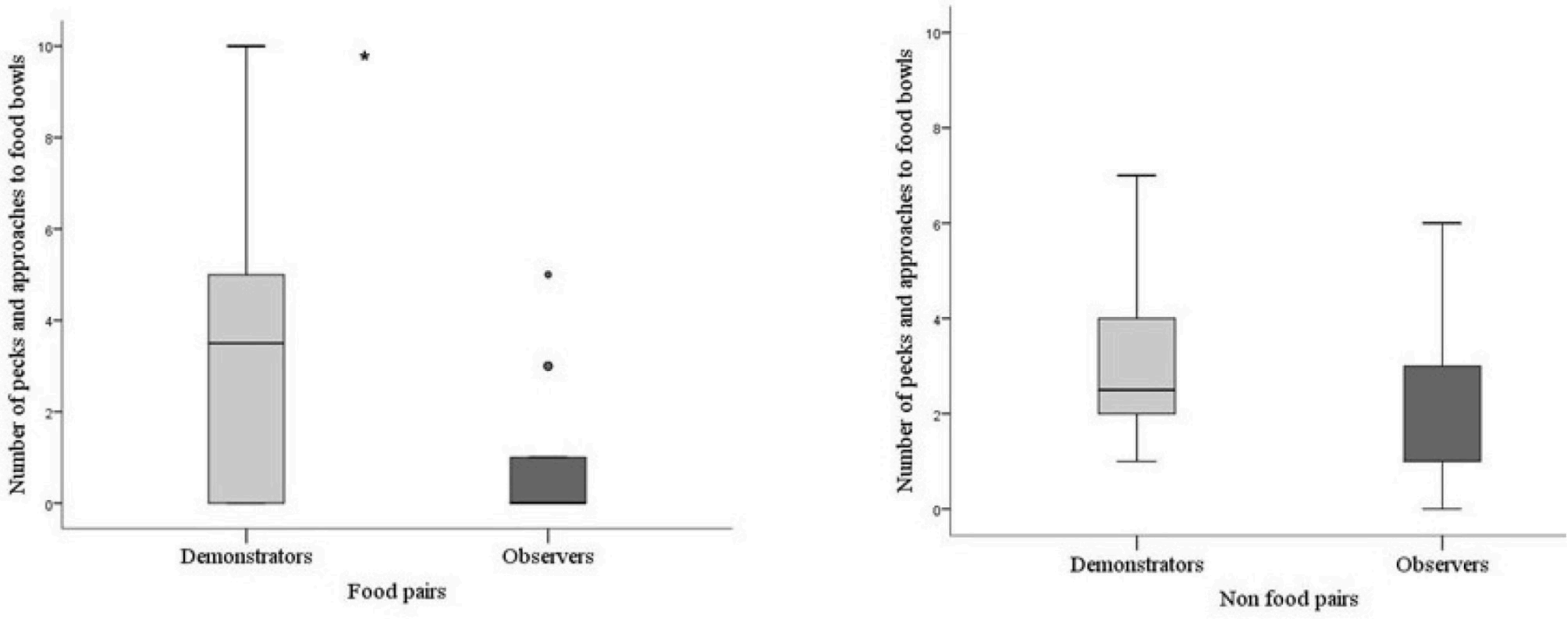
Pecks and approaches toward food bowls by demonstrators and observers in food and non-food pairs in batch 2. Thick horizontal lines indicates median nr of pecks, boxes span from the first to the third quartiles and the whiskers represent 95% confidence interval; outliers are denoted by dots. Demonstrators (*N* = 14) are the light gray bar and observers (*N* = 14) dark gray bar. The asterisk indicates a significant difference between treatments.

The demonstrators in batch 2 did not differ in how much they pecked the food bowls during testing depending on if they were together with the observer bird they formed a food pair together with or if they were with the observer they formed a non-food pair with (*P* = 0.842). This supports that the demonstrators behaved in the same way independently of whether they were in the role of being skilled or unskilled. In addition, the demonstrators also pecked the food bowls significantly more compared to the observers in food pairs (*P* = 0.024) and there was a tendency that this difference also persisted when they were paired in the non-food pairs (*P* = 0.087, Figure 4).

### Following behavior by observers

In batch 1 there was no significant difference between how often an observer bird followed the skilled (median ± IQR 1.5 ± 0–3.0) and unskilled bird respectively during the test (0.0 ± 0–0.25; Wilcoxon Signed Rank Test; *P* = 0.172, *N* = 10). However, in batch 2 the observer birds followed their food pair demonstrator significantly more compared to the non-food pair demonstrator (*P* = 0.005, N = 14; food pair 4.0 ± 1.0–8.0 (median ± IQR), non-food pair 0 ± 0–1.0).

### Comparing following behavior by demonstrators and observers

In batch 1 unskilled observers in general followed the other bird it was tested with more than the skilled demonstrator birds did (skilled 0.25 ± 0–0.5 (median ± IQR), unskilled 1.5 ± 0.125–2.87); Mann–Whitney *U*-test; *P* = 0.040, *N* = 24, 8 groups). But there was no significant difference when only the five groups that had not swopped demonstrators were included in the analyses (*P* = 0.165; skilled demonstrators 0.5 ± 0–0.75 and observers 1.5 ± 0–1.875).

**Figure 5:**
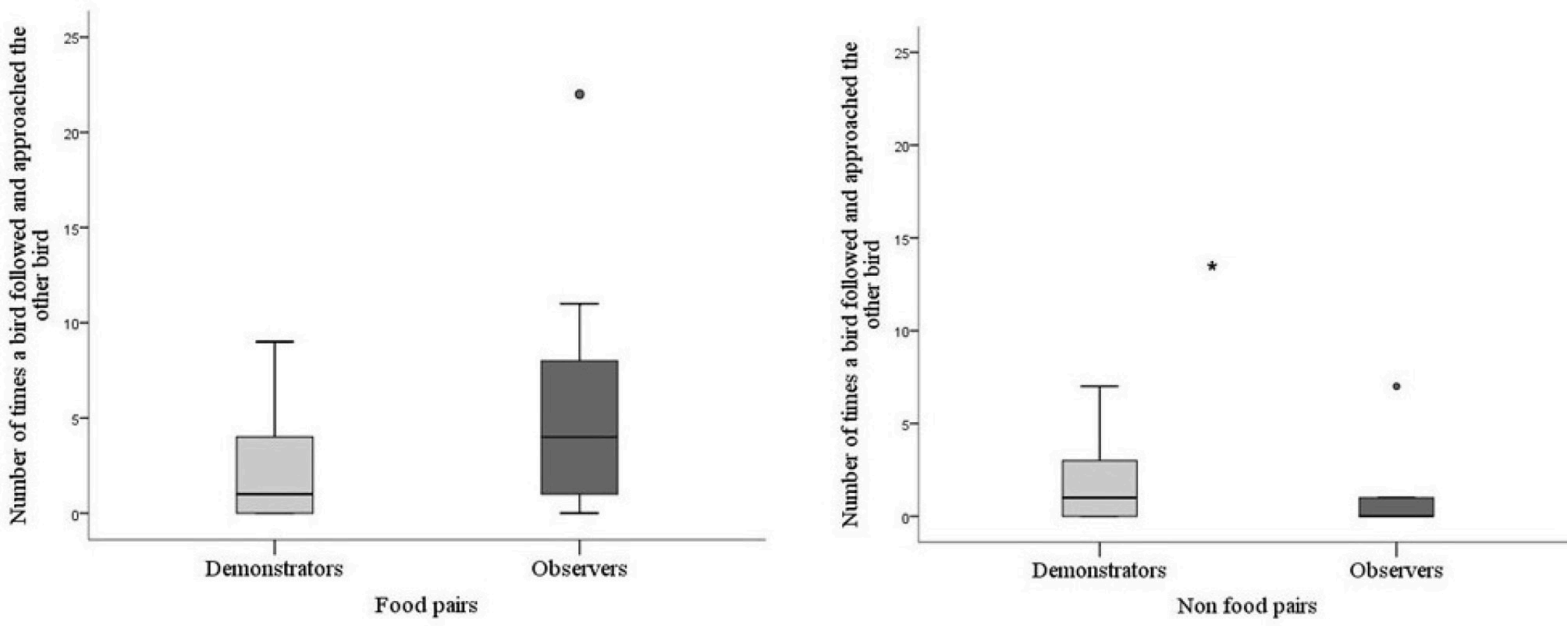
Number of times a bird followed and approached the other bird during testing for batch 2. Thick horizontal lines indicates median nr of pecks, boxes span from the first to the third quartiles and the whiskers represent 95% confidence interval; outliers are denoted by dots. Demonstrators (*N* = 14) are the light gray bar and observers (*N* = 14) dark gray bar. The asterisk indicates a significant difference between treatments.

For batch 2 a comparison was made between demonstrators and observers in their following behavior depending on whether they were in the food pair or in the non-food pair. Demonstrator birds tended to perform less following behavior (Mann-Whitney; *P* = 0.059) compared with observers when in their food pair. However, when they were in the non-food pair the demonstrator performed more following behavior compared with the observer (*P* = 0.021, Figure 5). This difference was due to a difference in the observers' behavior and not in the demonstrators. The observers showed more following behavior when together with the skilled demonstrator, whereas the demonstrators showed the same rate of following independently of which observer it was paired with (Wilcoxon Signed Rank Test; *P* = 0.857).

### Aggression

There were few cases of aggression between the birds in the tested pairs in batch 1. Out of the 15 paired tests aggression was only seen during three of these and avoidance in two (and only in the pairs that contained a skilled demonstrator). Therefore no statistical analyses were carried out on this behavior. However in batch 2, there was more aggression overall. There was significantly more aggression within food pairs compared to non-food pairs (Wilcoxon Signed Rank test; *P* = 0.024, Median ± IQR for food pair 0 ± 0–2 and non-food pair 0 ± 0–0). In almost all cases it was one of the birds within a pair that performed all aggressions; both birds never performed aggression.

## Discussion

During testing there was no evidence that the observers in batch 1 followed the skilled demonstrators more than the unskilled demonstrators. However, when the method of training was modified for batch 2, where they were trained in pairs rather than in trios, more following behavior was performed by the observers in the test when they were together with the skilled demonstrator compared with the unskilled demonstrator. Since the demonstrator birds pecked equally at the food bowls independently of whether they were in the food pair or in the non-food pair it is likely that it was not the actual behavior of the demonstrator in the test situation that affected the following behavior of the observer but the identity and skill of the demonstrator bird that led to that the observers followed them in this situation. This implies that the observer birds had made a connection between the skilled demonstrators and access to corn based on their previous experience of this bird and not on its behavior during the test. Giraldeau and Lefebvre (15) also found that in a flock of pigeons the scroungers were able to single out a producer. However, in that study the producer had been opening the tubes with food during the same session as the observations were carried out so it is possible that the scrounger's closer distance to the producer was a result of that bird's behavior (i.e., tube opening) in a very close time span. Thus in the present study we added to this information and found support for the hypothesis that laying hens are able to learn about the skill other individuals possess and use this information to get access to food.

The reason for the difference in following behavior between observers in batch 1 and 2 is probably that being paired only with one demonstrator made the role of the demonstrators more clear for the birds in batch 2. During training with batch 1 when there were three birds in the arena at the same time it happened that one of the unskilled birds reached the food source very quickly after the skilled demonstrator and the remaining bird might not have noticed who came first. Thus it is possible that it might have registered both the other birds as a cue to get food and thus the cue given from the demonstrator bird was not as consistent for observers in batch 1 as in batch 2.

Nicol and Pope (11) found no evidence that hens who only had observed a successful feeder were able to transfer this information into a different foraging context. One reason for the differences between their study and the present study could be that they tested their birds in a different context whereas in the present study the birds were tested in the same context as they had been trained. Another difference in the methods between the studies was that in the present study the demonstrator and observer birds could interact and feed together. One of the aims with the developed test method was that it should be a near to natural situation where interaction between individuals could take place since this has previously been shown to be an important factor for social learning to occur. For example (20) compared how young chicks were influenced in their choice of food after having interacted with one demonstrator or just having observed another one. They found that the demonstrators functioned as tutors in both situations, but the influence was more successful when they could interact together. Also (21) found that juvenile Canary birds husked seed sooner if they had interacted freely with a familiar adult. However, there is also a risk that social learning can be suppressed by reduced ability of a subdominant individual to get access to resources if they are together with a more dominant individual (10). This could have had some influence in our study, although the aim was to select birds that were neither the most dominant or subdominant members of the group, these differences existed and affected their performance. This or a more general fearfulness could be a reason for why some of the initially chosen demonstrators had to be replaced by others due to their lack of eating reliably from the bowls.

In general the demonstrator birds performed more pecking and approaches toward the food bowls than the observers. They also showed less following behavior toward the other bird which indicates a more independent behavior compared with the observers. This is supported by the finding that the demonstrators in batch 2 were not influenced by who they were paired with and did not use their companion as a cue to whether the food bowls would provide them with food or not. This suggests that for them the food bowls and their own behavior overshadowed the cue the observers might have given whether food would be available or not.

There was a higher rate of aggression between birds in batch 2 which could have been linked to the fact that the birds to a larger extent associated the other bird with getting access to food. It was rather clear that for some birds the following behavior had an aggressive component. Thus it is likely that they experienced it as a competitive situation since the food sources during training could be depleted and the corn seemed highly valuable to the birds. The hens participating in the test had been selected as being of an intermediate dominance status i.e., neither performing nor receiving the most of the aggressive encounters within the group in their home pen. The reason why most of the aggressions were performed by the demonstrators could be because by chance they happened to be more dominant or it might be a result of their experience of finding the corn themselves increased their motivation and competitive ability to defend the food patch. Although hens have been found to show social discrimination under the stress of competition (22) the correlation between peck order and aggression in a competitive food situation has been found to be low (23).

## Conclusion

The results in this study support the hypothesis that hens are able to learn that a certain individual can lead them to a food source. Whether the hens follow another hen or not in the test situation is based on the demonstrator bird's previous success in gaining access to food and not on its specific behavior at the time. Hence, this implies that hens are able to make informed decisions on whom to follow during foraging based not just on more indirect information, as for example the demonstrators dominance status, but also from more direct information on the success of another individual.

## Author contributions

AW designed the experiment, executed the practical work, analyzed the results and wrote the manuscript.

### Conflict of interest statement

The author declares that the research was conducted in the absence of any commercial or financial relationships that could be construed as a potential conflict of interest.
